# EEG Microstates and Psychosocial Stress During an Exchange Year

**DOI:** 10.1007/s10548-020-00806-0

**Published:** 2020-11-09

**Authors:** Nursija Kadier, Maria Stein, Thomas Koenig

**Affiliations:** 1 Translational Research Center, University Hospital of Psychiatry and Psychotherapy, University of Bern, Bern, Switzerland; 2 Department of Clinical Psychology and Psychotherapy, University of Bern, Bern, Switzerland

## Abstract

The well-known stress vulnerability model of psychosis assumes that psychotic episodes result from the coincidence of individual trait dispositions and triggering stressors. We thus hypothesized that a transient psychosocial stressor would not only increase the number of and stress caused by psychosis-like symptoms (like delusion-like symptoms or auditory hallucinations) in healthy subjects but also elicit changes in EEG microstates that have been related to the presence of psychotic symptoms in patients with schizophrenia. Considering a radical change of one’s psychosocial environment as a significant stressor, we analyzed psychotic symptoms and EEG microstate data in teenage exchange-students at an early and a later phase of their stay. The subjects experienced a small and transient, but significant increase of stress by psychosis-like symptoms. These changes in mental state were associated with increases in microstate class A, which has previously been related to unspecific stress. microstate classes C and D, which have consistently been found to be altered in patients with psychosis, were found unaffected by the time of the recording and the subjective stress experiences. Therefore, we conclude that microstate class A appears to be a psychosis independent and rather general correlate of psychosocial stress, whereas changes in microstate classes C and D seem to be more specifically tied to the presence of psychotic symptoms.

**Electronic supplementary material:**

The online version of this article (10.1007/s10548-020-00806-0) contains supplementary material, which is available to authorized users.

## Introduction

As schizophrenia is a very complex and relatively common psychiatric disorder, it is crucial to learn more about the causes that contribute to its emergence and manifestation. Genetically determined components constitute a pre-existing vulnerability factor, explaining approximately 50–80% of the variance in twin studies (Sullivan et al. 2003). Apart from the presence of a genetic vulnerability, the emergence of symptoms also requires triggering factors. One of the critical exogenous triggering factors for schizophrenia is psychosocial stress (Holtzman et al. 2013).

Accordingly, the well-known diathesis-stress model (Holtzman et al. 2013) that postulates an interaction between pre-existing vulnerability and psychosocial stress (i.e., life events) can lead to the emergence of psychosis. Indeed, it has been shown that in already psychotic patients, the experience of stressful life events increased the risk of exacerbated psychotic and depressive symptoms (Ventura et al. 2000; Van Winkel et al. 2008).

When searching to understand schizophrenia on a neurophysichological basis, a particular subset of EEG microstates was repeatedly found to be consistently altered in affected patients. These microstates are assumingly EEG correlates of large scale cortical synchronization patterns that effectively gate the flow of information among cortical networks (Michel and Koenig 2018): A specific class of microstates (class D) covered consistently less time in patients with schizophrenia than in healthy persons, whereas the time contribution of another microstate class (class C) was found to be increased (Rieger et al. 2016). Besides, the time covered by class A microstates was found to be increased in schizophrenic patients, but this was considered an unspecific effect as it was also found in patients with panic disorders (Kikuchi et al. 2011).

Based on these findings, we hypothesized that the typical alterations of microstates during psychotic states could also be observed in a population that lacked a pre-existing genetic vulnerability, but that was systematically exposed to a considerable amount of psychosocial stressors.

Teenage students who participate in an international student exchange year constitute such a population, as they need to disengage from their primary support group and habitual social environment, suffer frequent interpersonal misunderstandings, and may become isolated due to language barriers. However, this population typically adapts to its new socio-cultural environment after some time, reducing the amount of psychosocial stress they have to endure. Therefore, we analyzed EEG resting-state microstate data from adolescent international students spending an exchange year in Switzerland recorded during an early and about five months later time into their stay. Also, the presence of psychosis-like symptoms was quantified using the Community Assessment of Psychic Experiences (CAPE42, (Hanssen et al. 2003)) that has been developed to investigate these types of experiences specifically in healthy subjects. In brief, the CAPE42 is a self-report instrument about positive, negative, and depressive psychotic symptom dimensions and queries the frequency of such symptoms, and, if present, the amount of distress caused by them (see http://cape42.homestead.com/ for the questions). Answers to each question are given on a 4 step scale and combined into 6 separate sum scores (3 dimensions × (“amount of” vs. “distress caused”)).

Four specific hypotheses were examined:

1. Psychotic symptoms or the stress elicited by them decrease as the length of the exchange stay increases due to an adaptation to the new environment.
2. The percent time spent in class D microstate correlates negatively with the presence of psychotic symptoms, as a relative absence of microstate D has been considered as a risk factor for psychosis-like experiences.
3. The percent time spent in microstate class C correlates positively with the presence of psychotic symptoms.
4. The percent time spent in microstate class A correlates positively with distress caused by eventual psychotic symptoms.

## Results

**Fig. 1:**
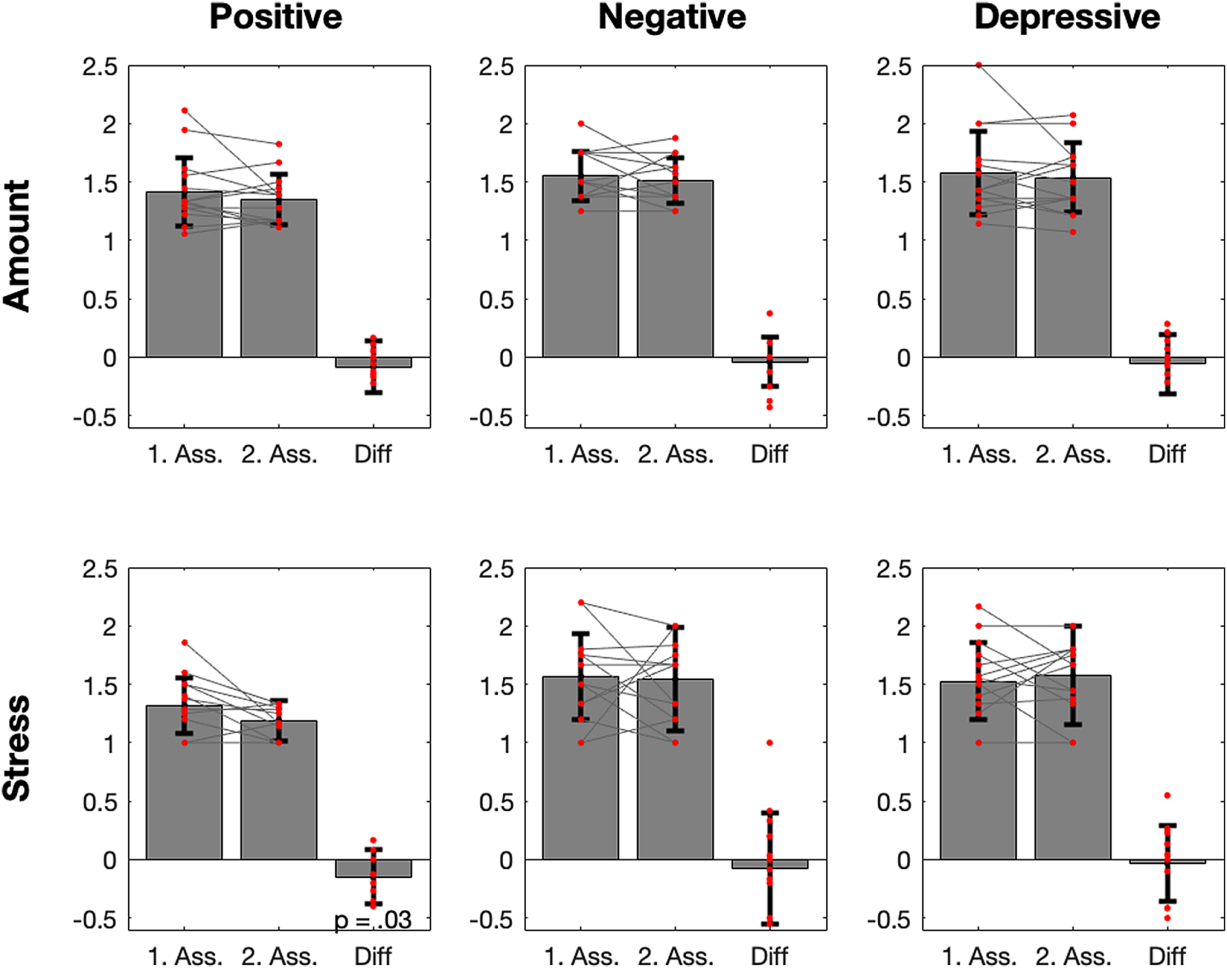
The mean amount of and stress experienced by the positive, negative, and depressive psychosis-like symptoms at the first and second assessment, as measured by the CAPE 42. Individual subjects are indicated by red dots and lined by lines. Error bars indicate the standard deviation

Overall, in the CAPE42 data, the number of psychotic experiences and the distress produced thereby were rather low already at the first assessment, which took place within the first three months in Switzerland: Mean values were around 1.5 on a scale from 1 to 4, suggesting that subjects experienced only little amounts of psychotic experiences and were hardly distressed by them. Nevertheless, all six sum scores showed a reduction in the second assessment five months later, compared to the first one (Fig. 1). In the case of distress produced by positive symptoms, this reduction was significant (p = .015, t = − 2.43, df = 13).

**Fig. 2:**
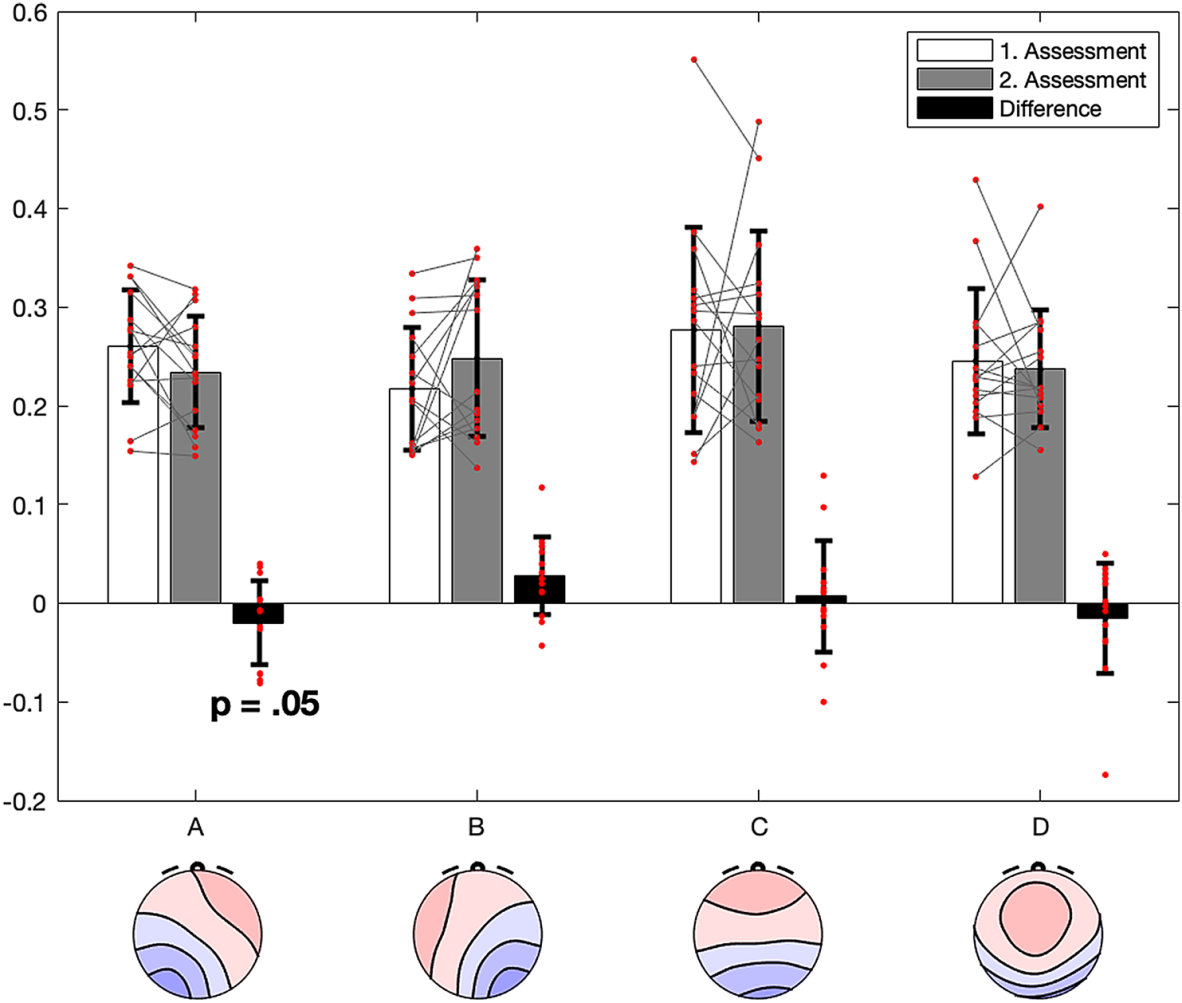
Changes in the contribution of microstate class A from the first to the second assessment, displayed in analogy to Fig. 1

Interestingly, the distress caused by positive symptoms at the first assessment was the only CAPE42 score that correlated with the overall pattern of microstate contribution (p = .048, Wilks lambda = 0.47, F = 3.77, df = 3,10). Further testing on the single microstate classes indicated that only microstate class A contribution was significantly associated with the amount of distress caused by positive symptoms (p = .031, r = .577, df = 13). As we had also found that the amount of distress caused by positive symptoms was reduced at the second assessment, we speculated that there should also be a consistent reduction of microstate class A contribution from the first to the second assessment. This was indeed the case (t = − 1.75, p = .05, df = 13), see also Fig. 2. In sum, our results yielded statistically significant support for hypotheses 1 and 4, but not for hypotheses 2 and 3. Complementary repeated measures models for microstate frequency and duration failed to reach significance.

## Discussion

Based upon the diathesis-stress model, the current study hypothesized that a temporally confined presence of psychosocial stressors would elicit an increase of psychotic experiences or lead to an increased amount of distress caused by such experiences (Holtzman et al. 2013). We used this situation to assess whether a transient stressor would also elicit changes of resting-state EEG microstates that were previously linked to schizophrenia (Rieger et al. 2016), or to microstate changes previously related to the presence of stress independently of the existence of psychotic symptoms. Fortunately, and not entirely unexpected, the psychosocial stressor’s impact on the participants’ mental health was only small. Nevertheless, our data gave evidence that the effect of this psychosocial stressor decreased throughout our participants’ stay, probably because of the acquisition of new culturally embedded psychosocial skills, coping strategies, and adaptive processes that took place between the first and the second assessment, thus tying these experiences to the particular situation at the beginning of an exchange year. Therefore, our naturalistic approach to study young and healthy exchange students appears to be a useful model to assess the neurophysiological effects of psychosocial stressors and related psychotic experiences.

The present study’s central finding is that the distress caused by stress-related psychotic experiences, not the frequency of such symptoms, was systematically correlated only with microstate class A: The more participants were distressed by their abnormal experiences, the more overall time they spent in this microstate. This correlation was seen at the first assessment when the students were likely to be yet less adapted and exposed to more psychosocial stress in their new environment. Consistently with this observation, the percent time spent in microstate class A also diminished systematically from the first to the second assessment. In contrast, no significant changes were found in microstate classes C and D, i.e., in those classes that were tied more explicitly to psychosis in previous studies. This may be due to a lack of statistical power, or, more interestingly, it may be that the hypothesized effects in microstate classes C and D are indeed specific to patients prone to schizophrenia, whereas changes in class A may relate to being stressed in general. Recent evidence in at-risk subjects or unaffected siblings indeed suggested that at least microstate class D may be linked to vulnerability rather than a stress marker (da Cruz et al. 2020). Our data, therefore, confirms the already previously made assumption that increased presence of microstate class A is a psychosis independent and rather general correlate of psychosocial stress (Kikuchi et al. 2011). In contrast, changes in microstate classes C and D seem to be unrelated to unspecific stressors and thus probably more specifically tied to the presence of psychotic symptoms.

## Electronic supplementary material

Below is the link to the electronic supplementary material.

Supplementary file1 (DOCX 23 KB)
