# Supplementary material for: EEG Microstates and Psychosocial Stress During an Exchange Year
Source: Brain Topogr. 2020 Nov 9;34(2):117–20. doi: 10.1007/s10548-020-00806-0 (PMC7892813; doi:10.1007/s10548-020-00806-0)
Supplement: Supplementary file 1 — Supplementary file1 (DOCX 23 KB) [file 10548_2020_806_MOESM1_ESM.docx]

**Material and Methods:**

Subjects

The test subjects consisted of 14 (10 female / 4 male) native English-speaking exchange students, with an average age of 17.2 years (Range: 16.0-18.5) years. All subjects lived in a local host-family and regularly attended the local high school. Subjects were assessed twice, the first assessment (day 1) was conducted within the subject's first three months in Switzerland, the second assessment (day 2)

5 months (range: 4–7) later. The data collection was part of a larger study on brain plasticity (Stein et al. 2006), had been approved by the local ethics committee, and agreed with the principles of the declaration of Helsinki. All subjects gave their informed consent at the beginning of the study.

Assessment of psychotic symptoms

To assess the amount of and distress experienced by psychotic experiences, participants filled out the “Community Assessment of Psychic Experiences” (CAPE42, (Hanssen et al. 2003)) questionnaire at the beginning of each assessment. The CAPE42 has 42 questions ranging from 1 (“Never”) to 4 (“Nearly always”) that assess, for each of the three dimensions of positive symptoms, negative symptoms, and depressive symptoms the frequency of and distress caused by particular symptoms. The corresponding 6 sum scores were computed for each subject and assessment.

EEG recording and analysis

After the participants had filled out the questionnaire, a resting state EEG was conducted. 74 silver/silver chloride electrodes were positioned at standard locations of the 10-10 system on the subject’s head. Resistance was kept under 10 kΩ. To monitor the artifacts, two additional electrodes were placed under the eyes. Cz served as the recording reference. The participants sat comfortably in a soundproofed room. The resting EEG was digitally recorded (250Hz sampling frequency) using an M&I BrainScope system. Task-free resting EEG data was recorded in two blocks each consisting of 20 seconds with open eyes, followed by 80 seconds with closed eyes.

The obtained EEGs were offline corrected for eye-movements using an ICA approach (Jung et al. 2000) and recomputed to average reference. The remaining artifacts were marked by visual inspection. Finally, the data was segmented into artifact-free 2-second epochs.

For the calculation of the microstate quantifiers, the epochs were filtered by 2-20 Hz, and the global field power (GFP) was calculated for each point in time. As the topography around the momentary maxima of the GFP stays stable, and mostly changes during the minima, only topographies with momentary maxima of the GFP were further analyzed. The data was then assigned to the 4 microstates topographies published by (Koenig et al. 2002), using a best-fitting criterion for the moments of GFP peaks, or a nearest-neighbor interpolation for the periods between GFP peaks. Single microstates were then defined as continuous time periods assigned to the same microstate class. No minimal length was set for a microstate. Individual quantifiers of microstates were created from the resulting assignments and averaged across epochs within-subject and assessment time. To limit the amount of multiple testing, only the most general feature of these profiles, namely, the mean percent overall time spent in each microstate class (“contribution”) was computed across epochs and within-subject and assessment and used to test the above hypotheses.

Statistical analysis

Differences in frequency of and distress caused by psychotic experiences between the first and the second assessment (hypothesis 1) were tested using one-tailed paired t-tests for each of the 6 sum scores of the CAPE42, expecting a reduction at the second assessment. To establish the significance of relationships between the CAPE42 sum scores and the microstate features with a relatively small number of tests, repeated measures models including all microstate classes were computed for each of the 6 sum scores of the CAPE42. Where these models across all microstate classes became significant, the significance of the relationship between each microstate classes contribution and the corresponding CAPE42 sum score was tested using Pearson correlation coefficients.

**References**

Hanssen M, Peeters F, Krabbendam L, et al (2003) How psychotic are individuals with non-psychotic disorders? Soc Psychiatry Psychiatr Epidemiol. https://doi.org/10.1007/s00127-003-0622-7

Jung TP, Makeig S, Humphries C, et al (2000) Removing electroencephalographic artifacts by blind source separation. Psychophysiology. https://doi.org/10.1017/S0048577200980259

Koenig T, Prichep L, Lehmann D, et al (2002) Millisecond by millisecond, year by year: Normative EEG microstates and developmental stages. Neuroimage 16:. https://doi.org/10.1006/nimg.2002.1070

Stein M, Dierks T, Brandeis D, et al (2006) Plasticity in the adult language system: A longitudinal electrophysiological study on second language learning. Neuroimage 33:. https://doi.org/10.1016/j.neuroimage.2006.07.008
